# Standardization of Trauma, General Surgical Morbidity and Mortality Conferences: Development and Dissemination of a “Toolkit” in Peru

**DOI:** 10.1007/s00268-022-06752-1

**Published:** 2022-10-10

**Authors:** Gabriela Zavala Wong, Manuel J. Rodriguez Castro, Eduardo Huaman Egoavil, Roberto Valderrama, Charles N. Mock, Juan J. Herrera-Matta, Gianni Aragon, Ryan Peterson, Ying Jin, Lacey N. LaGrone

**Affiliations:** 1 Alberto Hurtado School of Medicine, Universidad Peruana Cayetano Heredia, Av. Honorio Delgado 430, Lima, Peru; 2 Postdoc, University of Washington, 4333 Brooklyn Ave NE, Seattle, WA USA; 3 Research Division, Sociedad de Cirujanos Generales del Peru, Av. Arenales 2049, Lima, Peru; 4 Department of Surgery, Hospital Nacional Guillermo Almenara, Jr. Garcia Naranjo 840, Lima, Peru; 5 Department of Global Health, University of Washington, 1400 NE Campus Parkway, Seattle, WA USA; 6 Department of Surgery, Hospital de Policia, Av. Brasil 26, Lima, Peru; 7 Department of Surgery, Clinica Limatambo, Av. Republica de Panama 3606, Lima, Peru; 8 Department of Surgery, Clinica San Gabriel, Av. la Marina 2955, Lima, Peru; 9 Department of Biostatistics & Informatics, Colorado School of Public Health, University of Colorado Anschutz Medical Campus, 13001 East 17th, Aurora, CO USA; 10 Department of Surgery, University of Colorado, 2500 Rocky Mountain Ave, Loveland, CO USA

## Abstract

**Background:**

Morbidity and Mortality (M&M) conferences allow clinicians to review adverse events and identify areas for improvement. There are few reports of structured M&M conferences in low- and middle-income countries and no report of collaborative efforts to standardize them.

**Methods:**

The present study aims to gather general surgeons representing most of Peru’s urban surgical care and, in collaboration, with trauma quality improvement experts develop a M&M conferences toolkit with the expectation that its diffusion impacts their reported clinical practice. Fourteen general surgeons developed a toolkit as part of a working group under the auspices of the Peruvian General Surgery Society. After three years, we conducted an anonymous written questionnaire to follow-up previous observations of quality improvement practices.

**Results:**

A four-component toolkit was developed: Toolkit component #1: Conference logistics and case selection; Toolkit component #2: Documenting form; Toolkit component #3: Presentation template; and Toolkit component #4: Code of conduct. The toolkit was disseminated to 10 hospitals in 2016. Its effectiveness was evaluated by comparing the results of surveys on quality improvement practices conducted in 2016, before toolkit dissemination (101 respondents) and 2019 (105 respondents). Lower attendance was reported by surgeons in 2019. However, in 2019, participants more frequently described “improve the system” as the perceived objective of M&M conferences (70.5% vs. 38.6% in 2016; *p* < 0.001).

**Conclusion:**

We established a toolkit for the national dissemination of a standardized M&M conference. Three years following the initial assessment in Peru, we found similar practice patterns except for increased reporting of “system improvement” as the goal of M&M conferences.

## Introduction

Surgical diseases affect all ages, and injury, an emergency surgical problem, is the leading cause of death for people 45 years or younger and 90% of these occur in low–middle-income countries (LMICs), where 85% of the world’s population live [1–4]. It is estimated that 34–38% of all injury deaths in LMICs are preventable [3], if fatality rates among severely injured patients could resemble those in high-income countries (HICs) [3]. Furthermore, a study in 2003 compared trauma systems in HICs and LMICs and found that most of the staff in LMICs had less formal trauma training [4]. Trauma quality improvement programs (TQIPs) include structured assessment of patient care and patient outcomes to identify system improvements that may reduce preventable deaths and improve the processes of care. System referring to those services responsible that improve, maintain or restore health of individuals as per the World Health Organization (WHO) [5]**.**

The WHO and the International Association for Trauma Surgery and Intensive Care (IATSIC) have developed guidelines that summarize the essential components of trauma care across resource settings, including TQIPs. In a pilot study with 23 level I and II trauma centers, TQIPs were shown to be feasible and associated with a significant improvement in risk-adjusted mortality for blunt single- and multi-system trauma [1, 4]**.** Similarly, a WHO-IATSIC collaborative review showed that most TQIPs led to improvement in patient outcomes [6]. However, most of these data come from HICs [1, 6].

Morbidity and Mortality (M&M) conferences, a fundamental component of TQIPs, are opportunities for providers to review deaths and complications with a focus on system-wide improvement [7, 8]. While there are several reports of structured M&M conferences in HICs and a few in LMICs, there are no published reports of collaborative efforts to define a regional standard for M&M conferences with an accompanying toolkit [9, 10].

This project aimed to establish regional (Lima, Peru) consensus on a standard for surgical M&M conferences and facilitate diffusion of this standard via a practical, simple M&M “toolkit.” To determine the effects of passive diffusion of the toolkit by stakeholders in Lima (Peru’s capital city), we conducted serial evaluation through anonymous questionnaires to assess changes in M&M conferences practice in Peru prior to, and three years following, toolkit development.

## Materials and methods

This project represents “phase two” of a multi-phase project to implement quality M&M conferences in Peru. Phase one included a baseline assessment of existing practices in Peru and Latin America and a review of global implementation of the WHO TQIP guidelines [11–13]. It revealed standardized case selection criteria, documentation of M&M conclusions and a clear plan for follow-up to predict M&M conferences which are perceived to result in institutional change [11–13].

The second phase, presented in this manuscript, reflects an initial response to the data collected: development of a “toolkit” designed to be concise, self-explanatory and applicable to a diverse array of hospital and service types.

### Toolkit: development and dissemination

The Quality Chapter of the Peruvian General Surgery Society convened a working group. Individuals identified for the working group included those interested in quality improvement (QI) and having prior involvement with the Quality Chapter. Furthermore, the group was devised to include one or two general surgeons from each of the ten largest public, social security or military (non-pediatric) hospitals in Lima. Input was also provided by outside experts in trauma QI.

At the initial meeting, a summary of the relevant data from both the systematic review and the quantitative and qualitative assessment of QI practices in Latin America was presented to group participants. The group was then asked to reflect on these data and offer additional insights into particular areas for improvement in M&M conference practices in Lima. The working group met periodically over a six-week period. During this time, several of the working group members attended a course on trauma QI developed by the Panamerican Trauma Society.

The working group developed draft M&M toolkit based on examples from recent literature [9, 10]. This was revised iteratively. Working group member participants trialed an interim version of the toolkit components in their institutions. Their feedback was incorporated into its final revision. The final version was submitted to the Peruvian General Surgery Society leadership for ratification and dissemination.

Initial dissemination included passive diffusion by key stakeholders at the ten included hospitals in Lima. Three years after toolkit development, we conducted an interval assessment of TQIPs status in Lima.

### M&M conferences: assessment

The effect of the toolkit was evaluated by comparing the results of surveys on trauma quality improvement practices conducted in 2016 (before toolkit dissemination) and 2019. Baseline data (2016) for comparison came from a previously published survey [12]. This survey was repeated in 2019. In each case, the same questionnaire was used, the same interview procedures followed, and similar types of participants responded. We revisited nine out of 10 hospitals surveyed in 2016. The questionnaire included respondent demographics, hospital descriptors, self-reported QI practices at the respondent’s hospital and M&M conference characteristics. Respondents included surgeons, residents and medical students. Respondents were contacted by research assistants at the Peruvian General Surgery Society, at other conferences and at hospitals in Lima.

Results from 2016 and 2019 were compared. The R project for statistical computing (Statistical Computing, Vienna, Austria) was used for data analysis. We tested for significance using Fisher’s exact tests since all responses were categorical. Unadjusted p-values are presented together with *p*-values adjusted for multiple comparisons using Holm’s method [14].

Multivariable logistic regression was employed to assess which variables were associated with a reported institutional change resulting from M&M conferences. We included M&M conference frequency, whether the respondent reported: “improve the system” as the main objective of M&M conferences, perceived most valid source of medical knowledge, presence of standardized case selection criteria, absence of barriers to referring cases, note-taking during M&M conference, the proportion of case presentations missing information, number of attendings present, follow-up plan, opportunity to discuss errors, presence of trauma registry, survey year, and whether lack of interest or lack of staff education were listed as primary obstacles. “Number of M&M conferences per year” was the only variable not coded as dichotomous; these were derived from the question “How often do M&M conferences occur.” We employed a series of one-variable logistic regression models to evaluate each covariate’s unadjusted relationships and reported change. It is important to mention that the definition of “system” used during the development and dissemination of the toolkit was the one used by WHO [5], which was also aligned with the concept that local surgeons had about system. They understood “system” as in hospital surgical care of patients. Excluding prehospital care and hospital transport (both were not referenced in toolkit components) as these varies across Peru’s different regions, mainly because Peru lacks a unified and standardized trauma care, especially in the prehospital setting. Furthermore, during the toolkit development meetings there was a consistent education regarding M&M conference to be more about “system improvement” rather than blaming someone for a patient’s outcomes.

## Results

The following four toolkit components were developed by the working group.

### Toolkit component #1: guide for planning M&M conferences

**Fig. 1:**
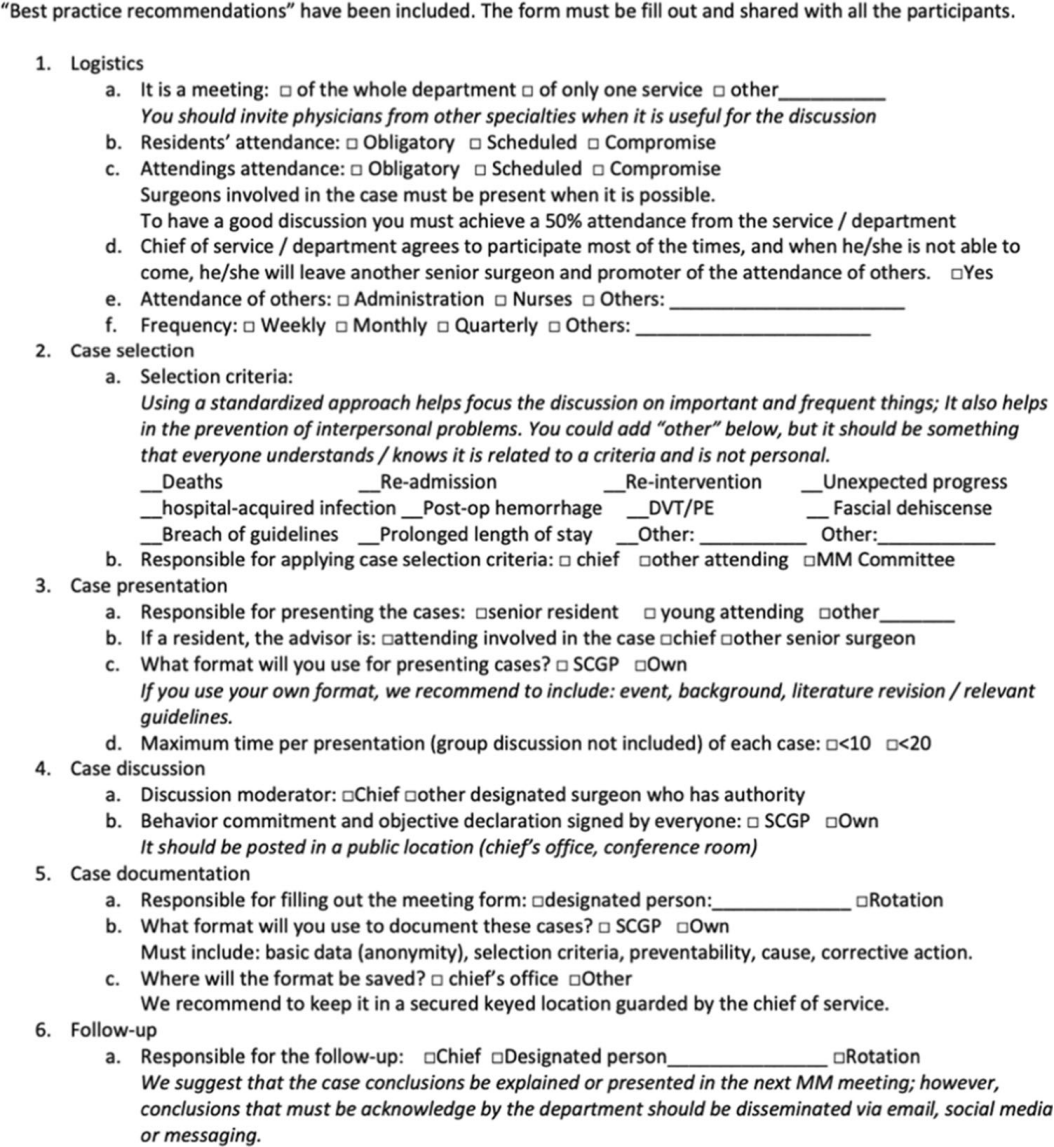
Guide for planning M&M conferences

The first toolkit component was developed to provide a guide, or checklist, to assist in planning a new or improving an existing M&M conference. This single-page document included conference logistics and case selection, presentation, discussion, documentation and follow-up. The guide includes evidence-based recommendations regarding M&M conference best practices and provides various options to allow the planner to modify the M&M processes according to what best serves their clinical and institutional scenario (Fig. 1).

### Toolkit component #2: form for documenting M&M conferences

**Fig. 2:**
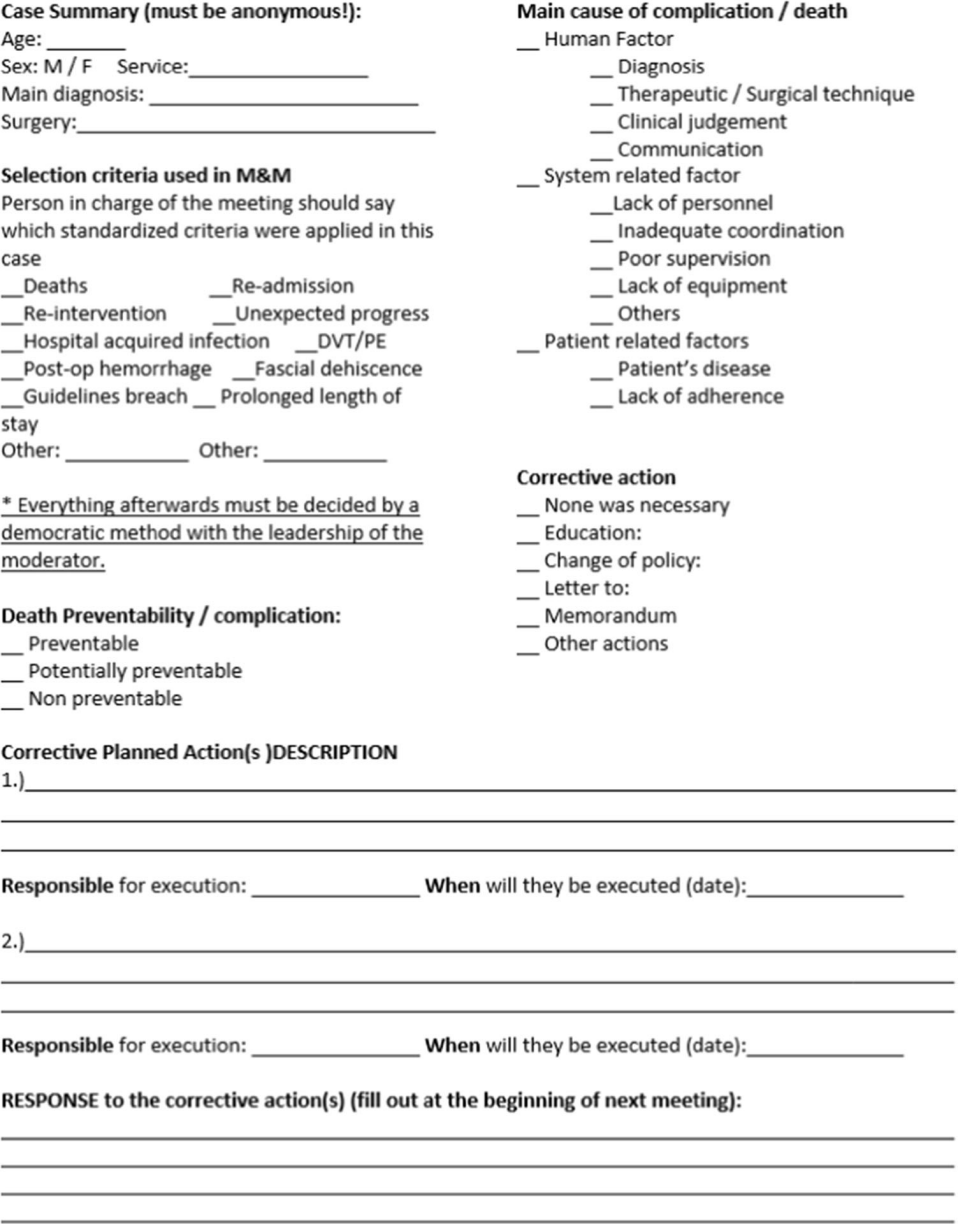
Form for documenting M&M conferences

This single-page document is designed to provide a record of the M&M case discussion to facilitate monitoring of trends in mortality and complications, to prompt conference participants to complete all essential components of an M&M discussion and to provide a place to document planned corrective actions to facilitate accountability and follow-up of those actions. The form included space to document anonymous patient description (age, sex, primary diagnosis), case selection criteria, an assessment of the preventability of the death or complication, an assessment of the primary cause of the death or complication, a detailed description of up to two planned corrective actions with a written designation of the person responsible for those actions and the timeline for completion (Fig. 2).

### Toolkit component #3: template for case presentations

**Fig. 3:**
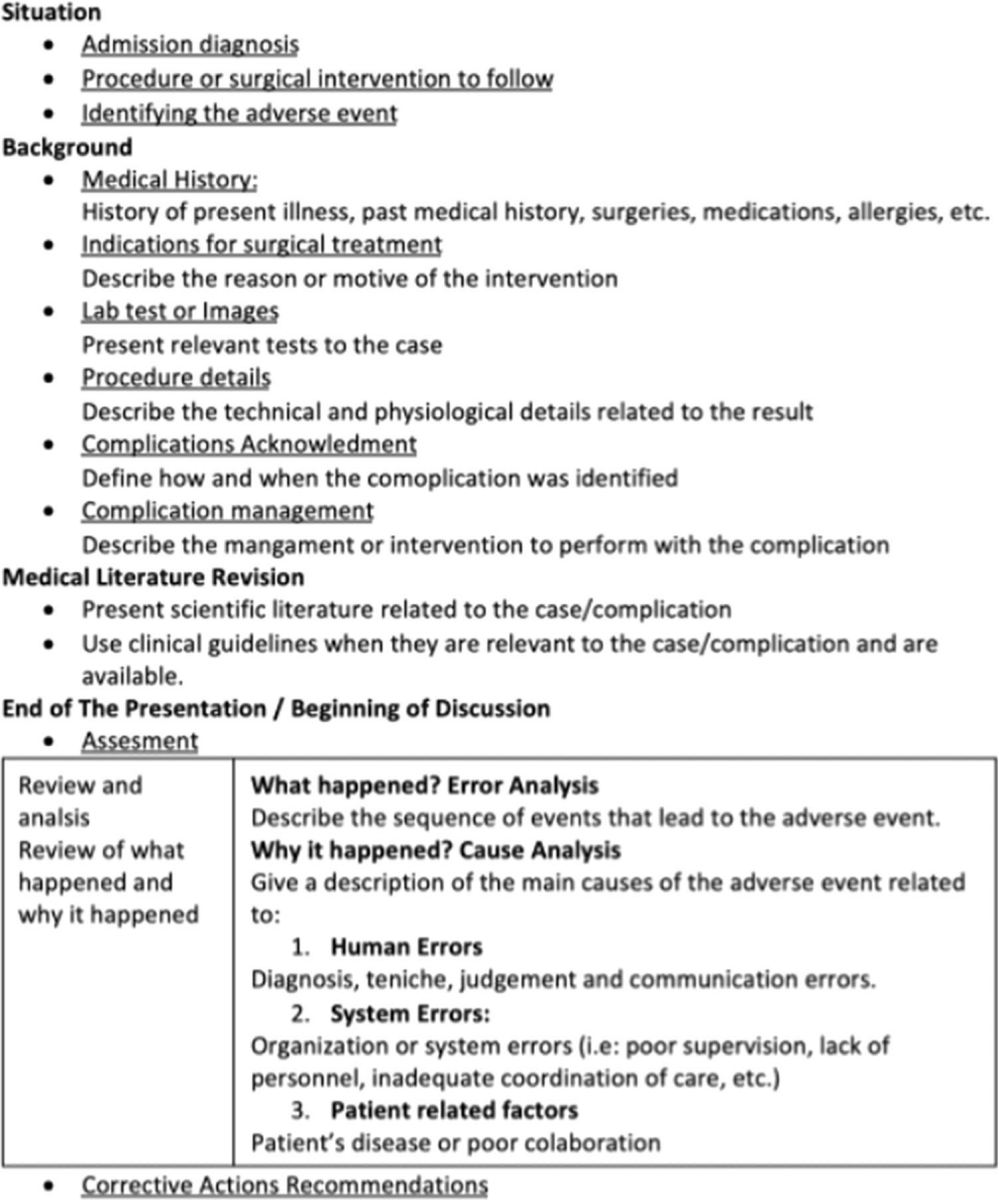
Case Presentation Model in Morbidity and Mortality meetings outline

A template was developed based on the Situation, Background, Assessment, Review [of literature] and Recommendations (SBAR) model proposed in an M&M guide developed by the Department of Surgery at Oregon Health and Science University [9]. It assists the junior clinician, often a resident, in selecting what information is salient in developing a concise case presentation, which includes all information necessary to inform a discussion of preventability and root cause. During the iterative toolkit development process, it was determined that the assessment and recommendations would be better left out of the presentation and included only in the discussion. Participants felt that residents did not have adequate experience with QI programs, root cause analysis and M&M conferences to make preliminary suggestions regarding assessment and recommendations. Furthermore, working group participants felt that the healthy interpersonal relations of the group would be more fully preserved if residents were not put in the position of offering assessment and recommendations for those senior to them (Fig. 3).

### Toolkit component #4: code of conduct

**Fig. 4:**
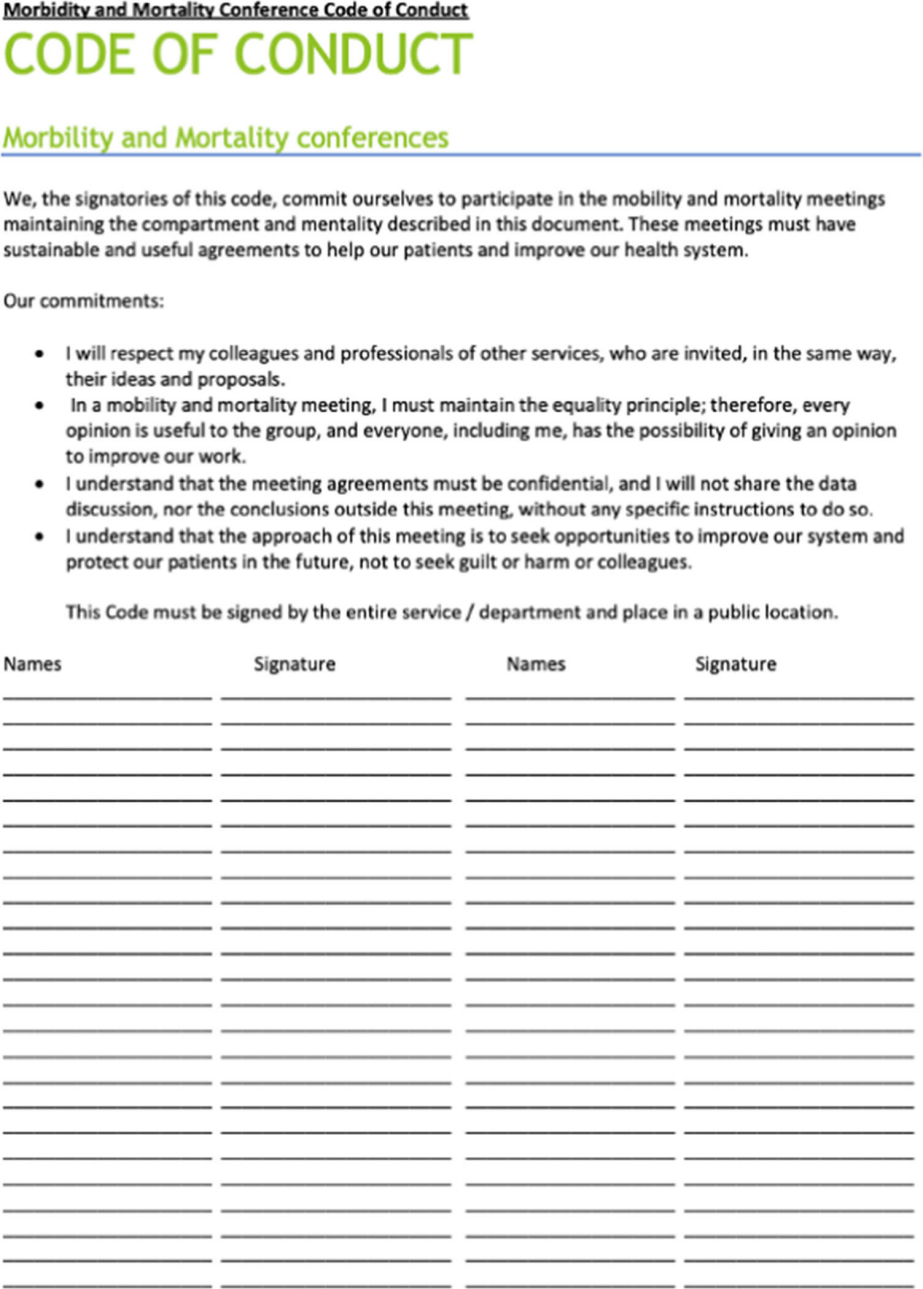
Code of conduct

Phase one research suggested that interpersonal conflict caused many failed attempts or non-sustainable M&M conferences in Lima. One suggested response to this common problem was the development of a “code of conduct” which would be signed by all participants and posted in a visible, central location (i.e., the chief’s office or the conference room). This code included participant commitment to mutual respect, openness to opinions of all conference participants, confidentiality and a focus on system improvement rather than searching for blame (Fig. 4).

### Interval assessment of TQIPs in Peru

A cross-sectional survey with 105 responses was conducted in 2019 and compared to the survey with 101 responses, which had been collected in 2016, three years prior to pre-toolkit development. Data from the two time periods reflected sampling of groups with similar demographics, but with fewer respondents from private hospitals in 2019, as compared to 2016 (16.1% vs. 2.1%, *p* = 0.02) and a higher proportion of respondents were medical students in 2019 as compared to 2016 (26.5% vs. 6.3%, *p* = 0.01) (Table 1). M&M conference characteristics were similar between the two time points, except for a trend toward lower reported attendance (Table 2). Additionally, there were more respondents in 2019 reporting “improve the system” as a perceived objective compared to 2016 (70.5% vs. 38.6%; *p* < 0.001) (Table 3).

**Table 1:** Demographics

	2016 (*N* = 101)	2019 (*N* = 105)	Unadjusted *p*	Adjusted p*
Location of hospital where you work			0.65	1
Urban	65 (98.5%)	101 (96.2%)		
Rural	1 (1.5%)	4 (3.8%)		
Missing	35	0		
Type of hospital where you work			< 0.001	0.02
Public	49 (79.0%)	71 (75.5%)		
Private	10 (16.1%)	2 (2.1%)		
EsSalud	2 (3.2%)	14 (14.9%)		
Other	1 (1.6%)	7 (7.4%)		
Missing	39	11		
Your clinical training			< 0.001	0.01
Attending	56 (88.9%)	61 (62.2%)		
Resident	2 (3.2%)	2 (2.0%)		
Medical student	4 (6.3%)	26 (26.5%)		
Other	1 (1.6%)	9 (9.2%)		
Missing	38	7		

*p-values adjusted for multiple comparisons using Holm’s method

**Table 2:** Characteristics of M&M conferences

	2016 (*N* = 101)	2019 (*N* = 105)	Unadjusted *p*	Adjusted p*	2016 Attendings (*N* = 57)	2019 Attendings (*N* = 61)	Unadjusted *p*	Adjusted p*
M&M conferences occur			0.25	1			0.67	1
No	4 (4.0%)	8 (8.2%)			3 (5.5%)	2 (3.4%)		
Yes	95 (96.0%)	89 (91.8%)			52 (94.5%)	56 (96.6%)		
Missing	2	8			1	3		
Frequency			0.5	1			0.33	1
Weekly	38 (38.4%)	35 (36.1%)			22 (40.0%)	16 (26.7%)		
Monthly	30 (30.3%)	24 (24.7%)			17 (30.9%)	17 (28.3%)		
Trimesterly	2 (2.0%)	4 (4.1%)			1 (1.8%)	4 (6.7%)		
Annually	4 (4.0%)	1 (1.0%)			0 (0.0%)	1 (1.7%)		
Rarely	21 (21.2%)	26 (26.8%)			12 (21.8%)	20 (33.3%)		
Never	4 (4.0%)	7 (7.2%)			3 (5.5%)	2 (3.3%)		
Missing	2	8			1	1		
Average number of attending physicians in attendance			< 0.001	0.03			0.01	0.39
1–2	0 (0.0%)	14 (16.1%)			0 (0.0%)	8 (15.1%)		
3–5	30 (42.3%)	25 (28.7%)			18 (35.3%)	11 (20.8%)		
5–10	27 (38.0%)	35 (40.2%)			22 (43.1%)	25 (47.2%)		
> 10	14 (19.7%)	13 (14.9%)			11 (21.6%)	9 (17.0%)		
Missing	30	18			5	8		

*p-values adjusted for multiple comparisons using Holm’s method

**Table 3:** Difference of each perceived objective of M&M conferences

	2016 (*N* = 101)	2019 (*N* = 105)	Unadjusted *p*	Adjusted p*	2016 Attendings (*N* = 57)	2019 Attendings (*N* = 61)	Unadjusted *p*	Adjusted p*
Decide on next steps in a patient’s treatment	17 (16.8%)	30 (28.6%)	0.05	1	10 (17.9%)	14 (23%)	0.65	1
Improve the system	39 (38.6%)	74 (70.5%)	< 0.001	< 0.001	29 (51.8%)	44 (72.1%)	0.04	1
Education	10 (9.9%)	14 (13.3%)	0.52	1	8 (14.3%)	4 (6.6%)	0.23	1
Assign blame	0 (0%)	4 (3.8%)	0.12	1	0 (0%)	3 (4.9%)	0.25	1

*p-values adjusted for multiple comparisons using Holm’s method

Significantly more medical students participated in 2019 compared to 2016. However, when data were analyzed only among attendings, no statistically significant differences were identified between the two time periods.

**Fig. 5:**
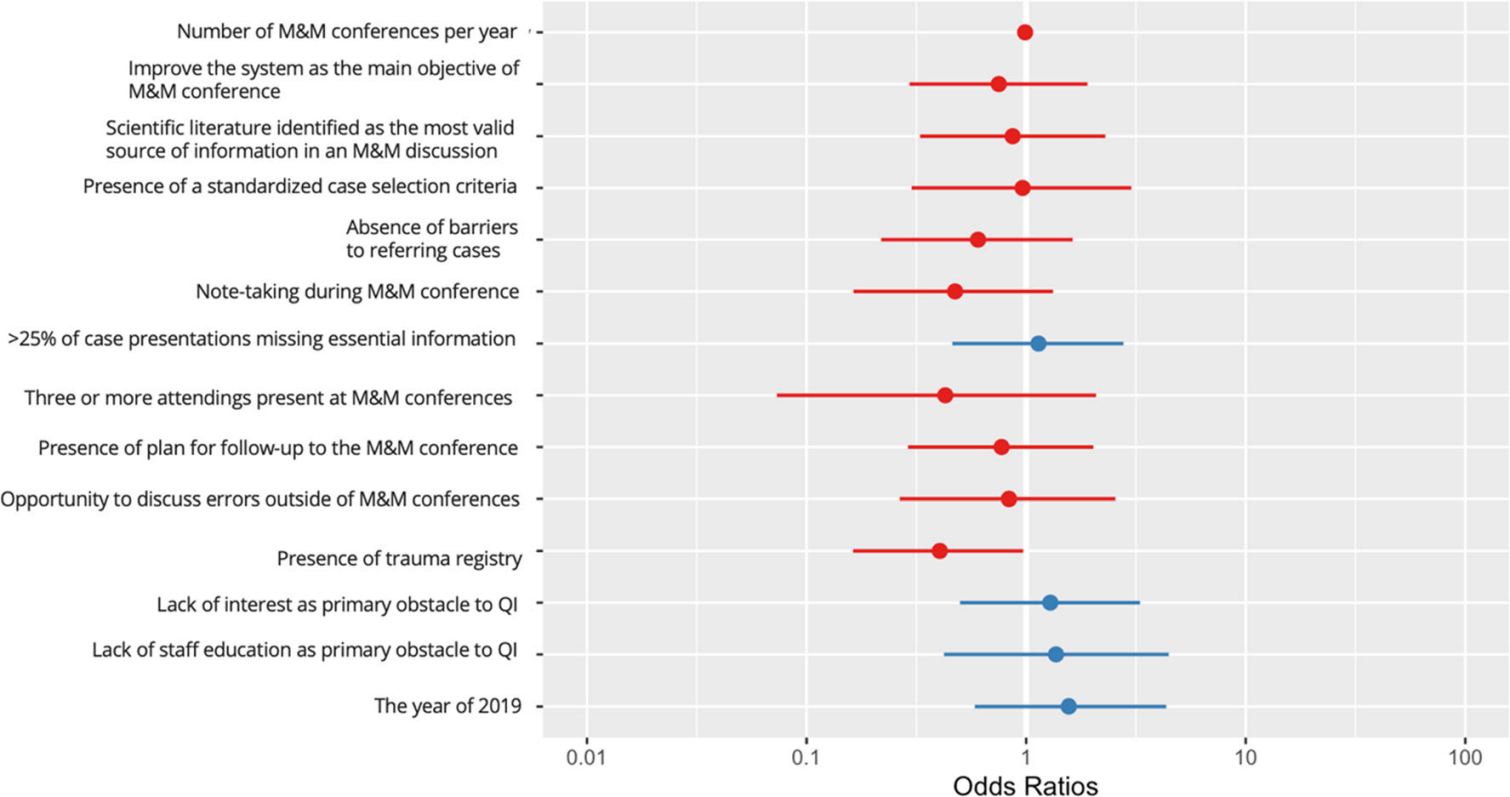
Multivariable logistic regression model predicting reported change as a result of the M&M conferences

Table 4, a multivariable model of the 2019 and 2016 Peruvian data, shows the only significant predictor of whether a respondent reported the presence of institutional change attributable to an M&M conference to be the presence of a trauma registry (*p* = 0.05) as compared to 2016 regression model [12]. This was associated with a decrease in the odds of reported change by a factor of 0.40 (95% CI: 0.16–0.97) (Fig. 5).

**Table 4:** Model output based on data of both years adjusted for year. Sample size of the adjusted model is 103

	Unadjusted relationships					Multivariate model			
	Sample size	Odds ratio	Confidence interval		*p* value	Odds ratio	Confidence interval		*p* value
			2.5%	97.5%			2.5%	97.5%	
Number of M&M conferences per year	145	0.981	0.967	0.995	0.009	0.989	0.969	1.009	0.301
Improve the system as the main objective of M&M conference	148	0.738	0.377	1.440	0.374	0.751	0.294	1.904	0.545
Scientific literature identified as the most valid source of information in an M&M discussion	147	0.529	0.267	1.036	0.065	0.868	0.329	2.297	0.774
Presence of a standardized case selection criteria	147	0.839	0.346	1.989	0.691	0.964	0.301	3.013	0.949
Absence of barriers to referring cases	139	0.504	0.246	1.016	0.057	0.604	0.219	1.629	0.321
Note-taking during M&M conference	148	0.539	0.272	1.051	0.072	0.475	0.163	1.325	0.159
> 25% of case presentations missing essential information	115	2.193	1.042	4.693	0.040	1.139	0.461	2.774	0.775
Three or more attendings present at M&M conferences	138	0.217	0.047	0.736	0.024	0.428	0.073	2.082	0.309
Presence of plan for follow-up to the M&M conference	147	0.728	0.365	1.438	0.362	0.773	0.290	2.022	0.601
Opportunity to discuss errors outside of M&M conferences	147	0.513	0.220	1.149	0.111	0.835	0.265	2.550	0.752
Presence of trauma registry	148	0.493	0.254	0.945	0.034	0.404	0.163	0.970	0.045
Lack of interest as primary obstacle to QI	148	2.157	1.105	4.276	0.025	1.289	0.500	3.302	0.596
Lack of staff education as primary obstacle to QI	148	0.903	0.370	2.171	0.819	1.368	0.422	4.462	0.599
The year of 2019	148	1.576	0.823	3.046	0.172	1.564	0.584	4.343	0.379

## Discussion

We sought to establish a regional standard for surgical and emergency M&M conferences and develop a simple toolkit to facilitate dissemination of that standard. The toolkit developed has significant overlap with those based at single institutions in high-income countries. However, the toolkit is distinct in its brevity—consisting of three single-page documents and a PowerPoint template, level of detail and comprehensiveness, self-explanatory and user-friendly format, and formation based on a consensus from individuals from more than eleven institutions and two continents.

This represents phase two of a three-phase endeavor—and is a response to the baseline assessment conducted in phase one. This toolkit aimed to address the three components associated with an effective M&M conference in previous studies [9]. These three components include modifiable but specific case selection criterion—included on both the planning guide and case documentation form; the case documentation form itself; and inclusion on the case documentation form of a written plan for follow-up. Phase three, and the next steps in this project, is the structured dissemination and implementation of the toolkit, with subsequent evaluation of provider perception of educational value, collegial environment of the M&M conference and its effect on patient outcomes. This toolkit may serve as a basis for similar processes in other countries—by which a national organization engages a group of stakeholders to modify the toolkit to fit the local culture and any additional evidence on M&M conference practices that may emerge.

In our follow-up assessment, we found similar practice patterns identified in 2019 as in 2016. Of the 31 variables included in our questionnaire, we found significant variations in demographics (different types of hospitals; *p* = 0.02), clinical training (*p* = 0.01), the average number of attending physicians (*p* = 0.03) and perceived objective of M&M conference (improve the system; *p* < 0.001) (Tables 1, 2 and 3), the latter of which may be explained by the increased number of medical students included in the surveys. No evidence of a change over these three years was found when analyzing only attending responses. However, the trend toward increased reporting of a focus on system-level changes persisted, but did not reach the level “significance” because as a subsample it has less power to find evidence against the null hypothesis.

The decrease in physicians’ attendance percent might also reflect a heightened standard for M&M conferences definition after the development of the M&M toolkit and exposure to these QI endeavors. In the multivariable regression model, the estimated odds ratio for trauma differed from resulted published in 2016 [12]. This might be explained by the fact that only Peruvian data was used for this regression model, thus making our sample smaller than in 2016 (which included other countries from the Andean region). Therefore, current sample had less power to reach statistical significance.

### Limitations

The utility of the toolkit itself may be limited by several factors. First, due to the logistical challenges of convening persons from broad geographic areas, the working group was comprised entirely of urban physicians. They worked at large academic and non-academic, public and private, institutions. Nonetheless, it may be that the toolkit has not addressed particular issues which would be relevant to a rural provider.

Additionally, this may also be affected by respondents being aware of research team observation as most of the surveys were complete in their presence (Hawthorne effect). Further, we used convenience sampling that may impact results.

This evaluation included responses from people who worked at a wide variety of institutions in Peru. The potential effect of the toolkit at the involved hospitals might be obscured by responses from hospitals to which the toolkit was not yet disseminated. Finally, it also important to highlight that there was not clear definition of “system” included in the survey, therefore that gave respondents room for interpretation, however, in a context familiar with “system” definition according to WHO guidelines and M&M conferences were more educational rather than punitive.

## Conclusions

A toolkit was developed to make trauma M&M conferences more standardized and effective. This was created with the collaboration of surgeons from the 10 major hospitals in the largest city in Peru. A general evaluation of trauma QI programs three years after this revealed minimal changes, except for an increase in the percent of respondents who felt that improving the system was the objective of M&M conferences. Next steps should be an active dissemination of the toolkit and a more in-depth evaluation of the extent its utilization. We recommend increased national-level prioritization of high-quality M&M conferences as a building block of TQIPs in Peru.
